# Anatomical Location of the Mesencephalic Locomotor Region and Its Possible Role in Locomotion, Posture, Cataplexy, and Parkinsonism

**DOI:** 10.3389/fneur.2015.00140

**Published:** 2015-06-24

**Authors:** David Sherman, Patrick M. Fuller, Jacob Marcus, Jun Yu, Ping Zhang, Nancy L. Chamberlin, Clifford B. Saper, Jun Lu

**Affiliations:** ^1^Department of Neurology, Beth Israel Deaconess Medical Center, Boston, MA, USA; ^2^Department of Medicine, Beth Israel Deaconess Medical Center, Boston, MA, USA; ^3^Department of Neurosurgery, Dalian Municipal Central Hospital, Dalian, China; ^4^Department of Endocrinology, Diabetes and Metabolism, The Second Hospital of Dalian Medical University, Dalian, China

**Keywords:** pontine motor, cataplexy, basal ganglia, sleep, dopamine

## Abstract

The mesencephalic (or midbrain) locomotor region (MLR) was first described in 1966 by Shik and colleagues, who demonstrated that electrical stimulation of this region induced locomotion in decerebrate (intercollicular transection) cats. The pedunculopontine tegmental nucleus (PPT) cholinergic neurons and midbrain extrapyramidal area (MEA) have been suggested to form the neuroanatomical basis for the MLR, but direct evidence for the role of these structures in locomotor behavior has been lacking. Here, we tested the hypothesis that the MLR is composed of non-cholinergic spinally projecting cells in the lateral pontine tegmentum. Our results showed that putative MLR neurons medial to the PPT and MEA in rats were non-cholinergic, glutamatergic, and express the orexin (hypocretin) type 2 receptors. Fos mapping correlated with motor behaviors revealed that the dorsal and ventral MLR are activated, respectively, in association with locomotion and an erect posture. Consistent with these findings, chemical stimulation of the dorsal MLR produced locomotion, whereas stimulation of the ventral MLR caused standing. Lesions of the MLR (dorsal and ventral regions together) resulted in cataplexy and episodic immobility of gait. Finally, trans-neuronal tracing with pseudorabies virus demonstrated disynaptic input to the MLR from the substantia nigra via the MEA. These findings offer a new perspective on the neuroanatomic basis of the MLR, and suggest that MLR dysfunction may contribute to the postural and gait abnormalities in Parkinsonism.

## Introduction

Work by Rhines and Magoun (1) provided the first indication that the reticular formation might participate in motor control by demonstrating that electrical stimulation of the reticular formation in decerebrate cats produced changes in muscle tone. Later work by Sprague and Chambers (2) showed that electrical stimulation of the reticular formation in freely moving, unanesthetized cats produced a variety of complex postural and motor behaviors, including circling, standing, and assuming sleep postures. In 1966, Shik and colleagues (3) showed that electrical stimulation within a confined region of the dorsal reticular formation (at the mesopontine junction) triggered walking and even galloping in otherwise immobile decerebrate cats. This confined area of the reticular formation was subsequently termed the mesencephalic locomotor region (MLR), and was considered a part of the cuneiform nucleus. Cuneiform nucleus is, however, an older term that included much of the mesopontine reticular formation. The term is often confused with the much smaller and better delineated nucleus of the same name used in modern literature, based upon the Berman cat atlas (4), which does not contain the MLR. To end this confusion, Skinner, Garcia-Rill, and colleagues refined the anatomical boundary of the MLR in rats to a more restricted region of the mesopontine reticular formation that included cholinergic neurons of the pedunculopontine tegmental nucleus (PPT or PPN) (5). Cholinergic PPT neurons have since been widely considered a key anatomical element of the anatomic and physiologic MLR (5).

Interestingly, however, lesions of neither the PPT nor the cuneiform nucleus block locomotion induced by stimulation of the lateral hypothalamus, a known input to the MLR (6, 7). Moreover, while cholinergic neurons of the PPT innervate the lower brainstem reticular formation, they provide few if any spinal projections (8). On the other hand, previous studies have revealed a band of non-cholinergic neurons just medial to the PPT that project directly to both the ventral horn of the spinal cord and to the ventromedial medulla (VMM) (8–11). Interestingly, this pontine cell group, which lies just ventral and medial to the PPT and ventral to the cuneiform nucleus, fits the general location originally proposed for the MLR. We have recently recorded from neurons in the lateral pontine tegmentum (LPT) by means of antidromic responses to spinal cord stimulation in freely moving animals, and found that the spinally projecting neurons fired in a bursting pattern with motor behavior while the non-spinally projecting neurons did not fire in association with motor behavior (12). Due to their connections with two crucial motor areas (i.e., the VMM and the spinal ventral horn), we hypothesized that these pontine spinally projecting neurons of the LPT are excellent candidates for the neuronal basis of the MLR.

The LPT contains substantial numbers of orexin-immunoreactive (-ir) terminals and high levels of them express the orexin 2 (OX2) receptor, whereas PPT cholinergic neurons express OX1 receptors (9, 13). Orexin neurons fire fastest during active locomotion (14, 15) and injection of an orexin-A agonist into the LPT region is reported to elicit locomotion in decerebrate cats (16), providing further support for the idea that the neurons in the LPT are modulated by orexin. Orexin is exclusively synthesized in the lateral hypothalamus (17), and studies in animals and humans have demonstrated that the disruption of orexin signaling (which is presumably excitatory) causes narcolepsy and cataplexy (sudden loss of muscle tone during wakefulness). Consistent with this, lesions of the LPT result in cataplexy in rats (9) and humans (18–22). These observations suggest that MLR neurons in the LPT may be important in both maintaining postural muscle tone against gravity and facilitating locomotion.

Both normal erect posture and gait initiation are impaired in patients with Parkinson’s disease (PD). And both the akinesia and gait freezing of PD have been attributed to inhibition of the PPT by overactive GABAergic neurons in the substantia nigra pars reticulata (SNr) and the internal segment of the globus pallidus (GPi in primates or endopeduncular nucleus in rats) (23). One recent study reported that deep brain stimulation (DBS) of the PPT region improved bradykinesia and gait in patients with PD (24). However, the pontine target of descending SNr/GPi input is in fact the midbrain extrapyramidal area (MEA), a region immediately medially adjacent to the cholinergic neurons of the PPT. This site, as well as the adjacent LPT reticulospinal neurons, may therefore have been activated by PPT-targeted DBS stimulation in the patients with PD. Thus, while the circuitry that causes the gait and postural disorders in PD remains poorly defined, it is possible that the ameliorative effects of DBS in PPT may depend most critically upon activation of motor circuitry in the MLR.

Given the foregoing, delineating the neuronal circuitry of the MLR may have significant implications for understanding fundamental issues of motor control and may provide a framework for understanding the circuit pathology of cataplexy and Parkinsonism. In the first part of the present study, we defined the MLR through the combined application of neuronal tracers, Fos mapping, chemical stimulation studies, and behavioral characterizations. In the second part of the study, we correlated chemical activation and lesions of the MLR with the occurrence of cataplexy and examined connections between the MLR and the orexinergic system. In the third part, we examine the connectivity of the MLR with basal ganglia output structures and explored the physiological and pathological implications of this circuitry. The results of the present study provide a new perspective on the neuroanatomic MLR, as well as provide a neuronal framework for understanding cataplexy and Parkinsonian akinesia and gait impairment.

## Results

### Pontine spinally projecting neurons contain glutamate and orexin 2 receptors

**Figure 1:**
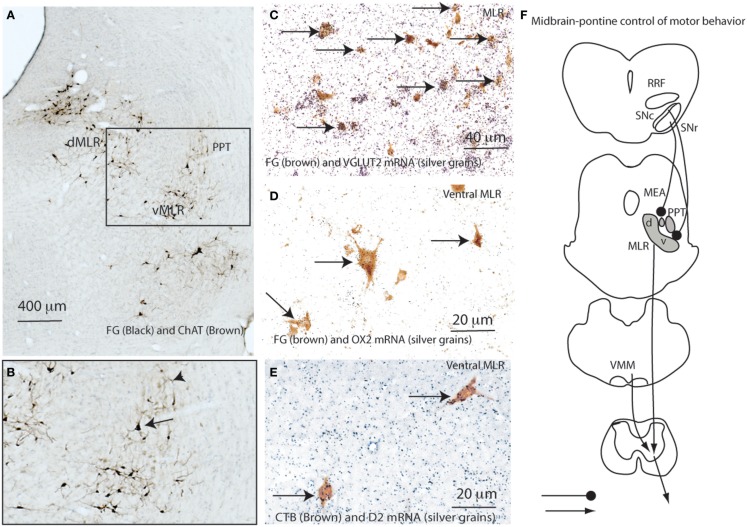
**Defining the neuroanatomic MLR**. **(A)** Retrogradely labeled (FG) neurons from the ventral horn (C8-T1 level) are seen within the dorsal (dMLR) and ventral (vMLR) components of the MLR. Pedunculopontine tegmental (PPT) neurons, labeled brown for ChAT, are seen lateral to the MLR **(A,B)**. The clear space between them represents the midbrain extrapyramidal area (MEA, also see Figure 7) **(A, B)**. Following spinal injections of FG, almost all FG-ir MLR neurons (brown) also express VGLUT2 mRNA (neurons overlaid by silver grains indicated by arrows) **(C)**. Similarly, about 50% of CTB-ir cells from the spinal cord (C8-T1) also contain OX2 mRNA (arrows) **(D)**. Less than 5% of reticulospinal neurons in the ventral MLR also contain D2 mRNA (arrows) **(E)**. **(F)** shows the proposed pontine motor neural circuitry.

We hypothesized that the potent locomotor effects of the MLR might be due to a direct spinal projection, and so defined a candidate cell population by injecting 20 nl of 10% Fluorogold (FG) into the spinal ventral horn and intermediate gray matter at the C8-T1 level in a series of 10 rats. FG was retrogradely transported into a large number of cells in the LPT, forming a strip running ventrally from the ventrolateral periaqueductal gray (vlPAG) to the region ventromedial to the PPT, with ipsilateral predominance. Dual labeling for FG and choline acetyltransferase (ChAT) immunoreactivity (Figures 1A,B) or for FG and vesicular glutamate transporter 2 (VGLUT2) mRNA by *in situ* hybridization showed that the FG-ir cells were exclusively non-cholinergic and almost all the FG-ir neurons were glutamatergic (Figures 1A–C). We have previously reported descending projections from the LPT mostly to the interneuron regions of the ventral horn (laminae VII and VIII) and less to motor neurons with a large number of appositions at the cervical level, fewer at the lumbar level, and fewest at the thoracic level (11). We hypothesized that these non-cholinergic and glutamatergic reticulospinal projections provide a marker for the neuronal population that constitutes the MLR.

### Chemical stimulation of the MLR induces locomotor and postural behaviors

**Figure 2:**
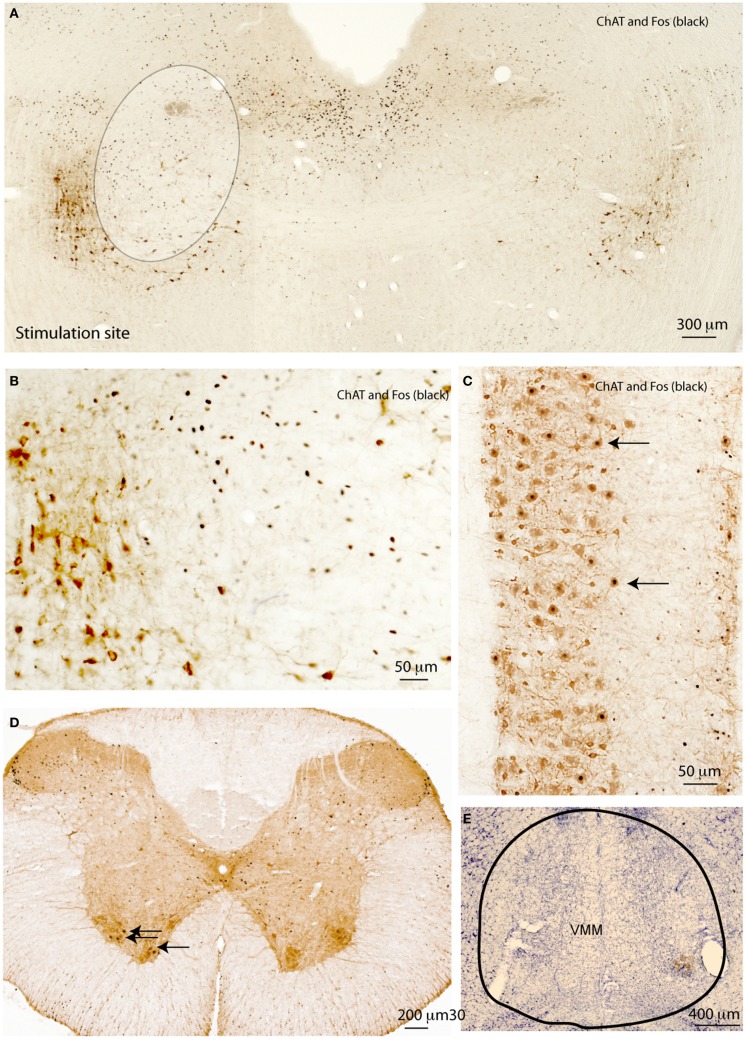
**Motor effects of MLR stimulation**. **(A)** Acute injection of 3 nl glutamate agonist (10%) ibotenic acid in the MLR region unilaterally induces contra-lateral rotation and c-Fos expression (black) in the MLR region, but not in PPT cholinergic neurons (brown, at lower left edge of circumscribed area) **(B)**. The same stimulation induces Fos in spinal motor neurons **(C)**. Fos is seen in spinal cord motor neurons ipsilateral to the stimulation site **(D)**. Lesions of the VMM **(E)** block neither circling behavior nor Fos expression in the motor ChAT-ir neurons induced by unilateral MLR stimulation.

To determine whether the cells in the region defined by the spinally projecting neurons were involved in locomotion, we studied whether focal stimulation of the LPT could induce any postural or locomotor behaviors. Under brief anesthesia with 2% isoflurane, rats received a stereotaxically placed injection of 3 nl of 10% ibotenic acid unilaterally in either the dorsal or ventral MLR (*n* = 5 at each site; Figure 2A). About 2 min after the termination of isoflurane in both groups, rats began circling toward the side contralateral to the injection site, and this behavior persisted for at least 3 h. At the end of 4 h, animals were reanesthetized with chloral hydrate and after perfusion with 10% formalin, the brains were removed and stained for Fos immunoreactivity and Nissl counterstained. Because of the short time after ibotenic acid injection and small volume (3 nl), there was no visible cellular injury and Fos-ir cells were visible in the ipsilateral but not the contralateral MLR region (Figure 2A). Fos was not expressed in the PPT cholinergic neurons (Figure 2B). In the spinal cord, Fos was seen in ChAT-ir motor neurons in the ventral horn ipsilateral but not contralateral to the stimulation site (Figures 2C,D). The stimulation of ipsilateral spinal motor neurons likely caused contralateral rotation.

Bilateral stimulation of the MLR region with 3 nl of ibotenic acid on each side caused continuous rhythmic walking. Animals (*n* = 5) would walk forward at a deliberate, but unhurried pace, until they reached a wall, at which point they would stand up against the wall. Animals also engaged in rhythmic whisker movement and vocalization.

### MLR control of motor behaviors does not depend on the ventromedial medulla

It has been hypothesized that the MLR may activate locomotion via projections to the ventromedial medullary reticular formation (11, 25). Specifically, Kinjo and colleagues identified PPT cholinergic inputs relaying information to the VMM (25). Here, we asked whether the MLR-VMM connection makes an important contribution to locomotor behavior. To address this, we first lesioned the ventral medullary reticular formation using injections of orexin-saporin (OX-SAP, 180 μg) (Figure 2E). These lesions extended in the rostral-caudal direction approximately from AP = −12.0 to −14.0 mm and from the midline 2.0 mm laterally (26). After 2 weeks of recovery, the rats received unilateral (3 nl) or bilateral (3 nl each site) injections of ibotenic acid into the MLR (*n* = 5 per group). These rats demonstrated patterns of circling behavior (unilateral injections) or walking behavior (bilateral injections) similar to the control rats, despite almost complete absence of neurons in the ventromedial medullary reticular formation (Figure 2E). In addition, Fos was seen in ventral horn motor neurons ipsilateral to the ibotenic acid injection sites in the lesioned rats. These results are consistent with earlier observations that chemical stimulation of the ventral medulla mainly resulted in reduction, not augmentation of muscle tone (27), indicating that the MLR activation of locomotion does not depend upon a relay to the ventral medulla.

### Activation of c-Fos expression by neurons in the MLR during locomotion and upright posture

**Figure 3:**
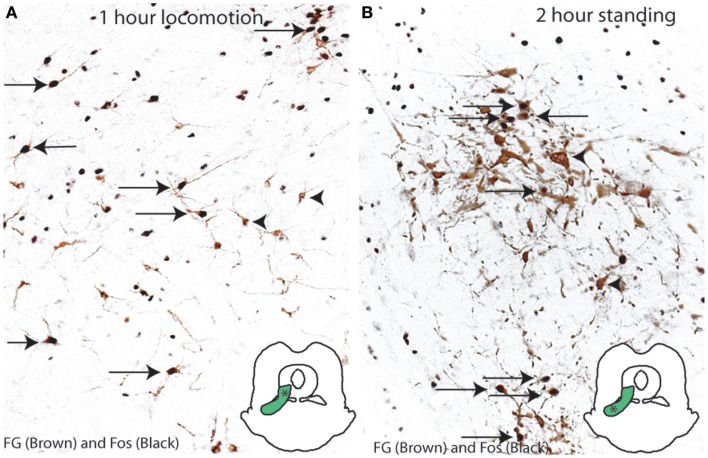
**Activation of the MLR neurons in motor behaviors**. **(A)** Locomotion (on a rotarod for 1 h) induces c-Fos expression in the dorsal MLR but not the ventral MLR. **(B)** Standing posture (2 h) induces c-Fos in the ventral MLR but not the dorsal MLR.

In order to determine which neurons in the MLR region are activated during normal gait and posture, we examined Fos expression in the LPT reticulospinal neurons of rats after locomotor or postural behaviors. Animals received an injection of FG into the C8-T1 level of the spinal cord, and after recovery, were compelled to walk on a rotorod (*n* = 5, speed of 8.0 RPM) for 1 h or to stand (*n* = 5, restrained vertically in a mesh cylinder) for 2 h, in order to mimic locomotor and postural activity seen after injections of ibotenic acid into the LPT. Reticulospinal cells were labeled for FG (brown) and for Fos (black) by immunohistochemistry in rats (*n* = 5/group). We found that, after performing a locomotor activity, Fos was expressed in 67.6 ± 6.8% of the FG-ir neurons in the dorsal group and none of the FG-ir neurons in the ventral group (Figure 3A). In contrast, following postural activity, Fos expression was displayed in 40.6 ± 3.3% of the FG-ir neurons in the ventral group of the MLR and none in the dorsal group (Figure 3B). Thus, the activity of spinally projecting neurons in the dorsal MLR appears to be associated with locomotion, and in the ventral MLR with upright posture.

### Lesions of the MLR produce cataplexy and immobility attacks

Cataplexy is a disorder in which patients with narcolepsy intermittently lose muscle tone and are unable to move during wakefulness. This atonia is similar to that seen in rapid eye movement (REM) sleep, but occurs during awake. We had previously hypothesized that the LPT region, which includes the spinally projecting neurons, has a role in generating muscle tone, and that it is normally inhibited during REM sleep, but that neurons in the LPT might themselves become inhibited during narcolepsy, thus allowing atonia (9). To further define the role the MLR neurons might play in mediating cataplexy, we first determined whether the spinally projecting MLR neurons contained the OX2 receptor for orexin (or hypocretin), the peptide neurotransmitter whose signaling is lost in narcoleptic animals and humans. We injected CTB into the ventral horn of the spinal cord at the C8-T1 level, and combined this with *in situ* hybridization for OX2 receptor mRNA in two rats. We found that many CTB-ir neurons in the dorsal MLR (44%) and ventral MLR (55%) expressed high levels of the OX2R mRNA (Figure 1D).

**Figure 4:**
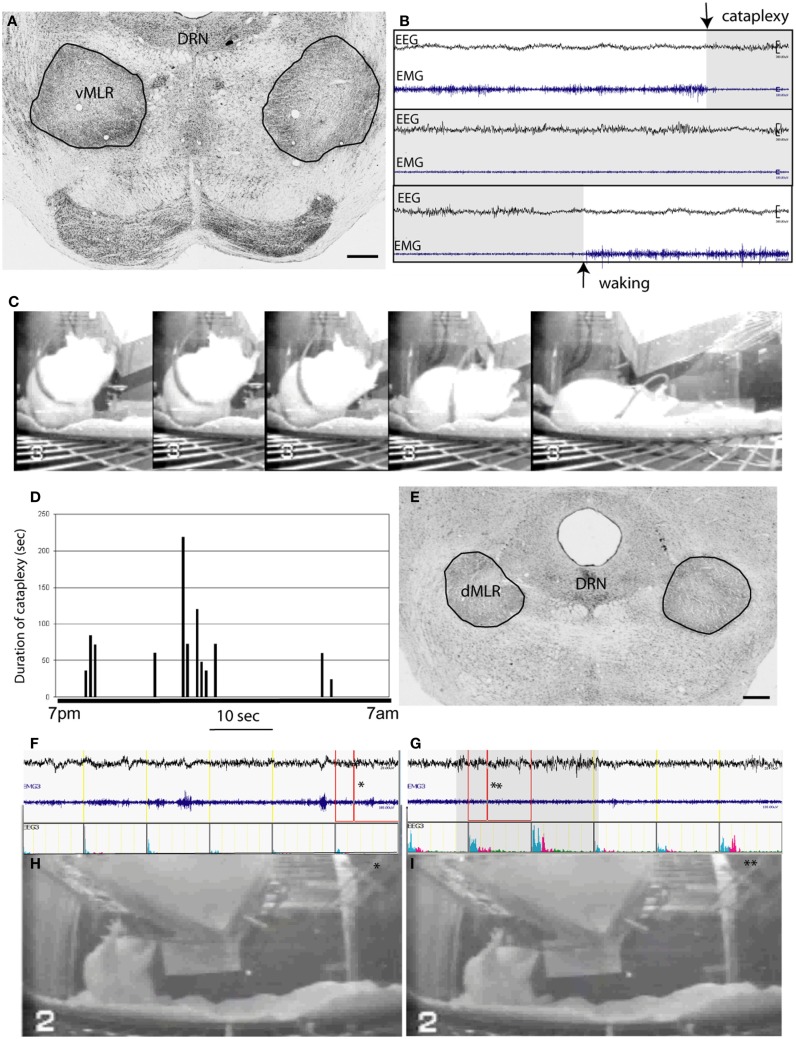
**OX-SAP lesions of the MLR produce cataplexy and complete akinesia**. **(A)** shows lesions (circled) including the vMLR but not the dMLR. A cataplexy attack caused by vMLR lesions, characterized by entering directly from wakefulness to a state of desynchronized EEG and atonia, is seen at the arrow in **(B)**. The animal is seen in a series of video images **(C)** showing an attack that begins while the animal is feeding, and results in him collapsing on the floor of the cage. The EEG resembles waking EEG not REM sleep EEG (high theta). **(D)** shows the duration and timing of a series of cataplexy attacks that occur during the dark period (7 p.m.–7 a.m.). Attack episodes vary from 20 to over 200 s in length. Lesions of the dMLR **(E)** produce freezing attacks or complete akinesia, in which the animal maintains the same posture throughout, despite being awake in the early part of the attack [EEG in **(F)** shows an awake pattern, photo in **(H)** is at time point indicated by asterisk] but falling asleep while frozen [EEG in **(G)** shows sleeping EEG, photo in **(I)** is at the time point indicated by the asterisk]. The bar shows duration of sleep EEG during the attack.

We next placed lesions in the region of the MLR spinally projecting neurons using OX-SAP, a toxin that damages neurons, presumably by binding to the OX2 receptor. OX-SAP killed MLR and non-MLR neurons within 7–10 days, while selectively sparing nearby PPT cholinergic neurons (28). Consistent with our earlier findings following ibotenic acid lesions in the LPT region (9), bilateral OX-SAP lesions of the ventral MLR (*n* = 25) produced frequent cataplectic attacks, on average 10 per night, although certain cases with particularly large lesions (Figure 4A) exhibited about 15 per night with an average duration of about 80 s each (Figures 4B–D). Cataplectic attacks often occurred during feeding behavior and were characterized by a sudden drop in muscle tone, measured by electromyogram (EMG), during a period of wakefulness as identified by electroencephalogram (EEG). During these attacks, rats frequently experienced postural collapse (Figure 4C). When these bouts of paralysis lasted more than 30 s, EEG desynchronization was often seen, with a gradual increase in theta power.

Lesions targeting the dorsal part of the MLR caused immobility attacks lasting up to 30 min during which animals remained standing in one place (Figures 4E,F). The initial waking EEG would gradually slow into the range of a NREM sleep pattern about 15 s after the onset of paralysis. Often wake and sleep EEG alternated during the attacks. Although we only recorded EMG in cervical muscles, which showed a low level of activity during these attacks, it appeared that overall postural tone was diminished (Figures 4F,G), because animals leaning against a cage wall or feeder would often slide downward, but not fall suddenly to the floor of the cage (Figures 4H,I), as happens in cataplexy.

### Anatomical connections of the substantia nigra pars reticulata and pars compacta with the MLR

Previous research has found that basal ganglia output from the SNr and GPi of the basal ganglia targets the area immediately medial to the PPT cholinergic group, a structure that was identified by Rye and colleagues (29) as the MEA. AS the MEA appears adjacent or may even overlap the MLR and PPT, we wanted to explore the relationship between the descending nigral (SNr) output systems, MLR, and PPT.

**Figure 5:**
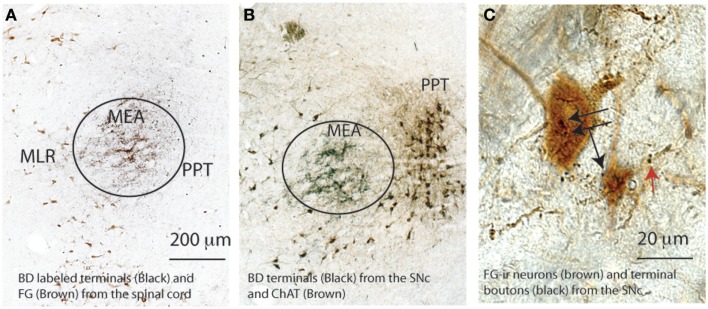
**Spatial relationship of the MLR, MEA, and PPT**. The MEA marked by black terminals of a biotinylated dextran (BD) injected into the SNr in both **(A,B)** situates lateral to the MLR marked brown by FG from the spinal cord in **(A)** and medial to the PPT, marked brown by ChAT-ir neurons in **(B)**. The ventral MLR (FG) neurons (black arrows) and their dendrites (red arrows) are apposed by SNc terminal boutons **(C)**.

We first labeled the descending projection from the SNr using simultaneous injections of anterograde tracer biotinylated dextran amine (BD, 6 nl) into the SNr and FG into the ventral horn (C8-T1 level) to reveal the MLR spinally projecting neurons (*n* = 3). We demonstrated that the BD-labeled terminals from the SNr targeted the MEA, which was just lateral to the dorsal MLR neurons and dorsal to the ventral MLR neurons (Figures 5A,B). Consistent with previous descriptions by Wainer, Rye, and colleagues (29, 30), the MEA was medial to the PPT, and thus, sandwiched between the MLR and PPT (Figures 5A,B). BD injected in the SNc terminated on the ventral MLR neurons (Figure 5C).

To further determine whether SNc neurons projecting to the vMLR were dopaminergic or non-dopaminergic, we injected CTB into the ventral MLR region in four rats, and then stained sections through the SNc immunohistochemically for TH and CTB. To our surprise, only a few of the CTB-ir neurons (<10%) in the SNc were TH positive. As the SNc contains both dopaminergic and GABAergic neurons, it is likely that the non-dopaminergic neurons were GABAergic. Consistent with this finding, few MLR neurons that were retrogradely labeled with FG after spinal cord injections (<5%) showed *in situ* hybridization for D2 receptor mRNA (Figure 1E).

To dissect the neural pathway of the MLR in the spinal cord motor system, we injected pseudorabies virus (PRV) expressing green fluorescence protein (PRV-GFP; a retrograde transneuronal tracer) into the soleus (ankle extensor) or anterior tibial (ankle flexor) muscles. Rats were perfused at 120 h (*n* = 2 per muscle), 168 h (*n* = 2 per muscle), and 192 h (*n* = 2 per muscle) after the PRV-GFP injections.

At 120 h, very few PRV-GFP retrogradely labeled neurons were observed in the VMM from rostral to caudal level after injections into either the flexor or extensor muscle (Table 1).

**Table 1:** **PRV-GFP-ir neurons (section/injection side) in the mesopontine-medulla motor circuitry**.

	Flexor (tibialis)			Extensor (soleus)		
	120 h (*n* = 2)	168 h (*n* = 2)	196 h (*n* = 2)	120 h (*n* = 2)	168 h (*n* = 2)	196 h* (*n* = 2)
RVM	*	**	***	*	*	***
CMM	*	**	***	*	*	***
vMLR	–	**	*****	–	*	*****
dMLR	–	****	*****	–	–	****
MEA	–	**	**	–	–	*
SNc	–	–	–	–	*	***
SNr	–	–	*****	–	–	–
RRF	–	*	**	–	*	**
LDT	–	–	–	–	–	*
PPT	–	–	–	–	–	–

*1–10 cells; **10–20 cells; ***20–30 cells; ****30–40 cells; *****40–50 cells.

**Figure 6:**
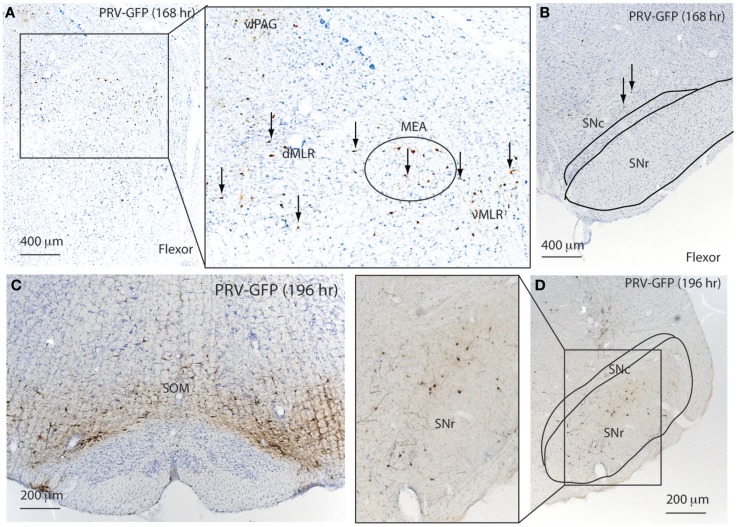
**Temporal and spatial distribution of PRV-GFP from the tibialis anterior (TA; flexor) muscle**. At 168 h after PRV-GFP injections in TA, PRV-GFP preferentially labels the PAG, MLR, and MEA **(A)** as well as the VMM, but not the SNr **(B)**. After 196 h, the PRV labeling from the TA shows more extensive labeling in the VMM **(C)** and SNr **(D)**.

At 168 h after flexor PRV injection, PRV-GFP further labeled the VMM, and began to appear in the PAG and entire MLR as well as the MEA (Figure 6; Table 1). However, no PRV was seen in SNr or SNc. At 196 h after flexor PRV-GFP injections, PRV-GFP was highly expressed in the VMM, vlPAG, MEA, MLR, and RRF. PRV was seen in SNr but not SNc neurons (Figure 6; Table 1). At 192 h, PRV-GFP expression in these regions was further enhanced. However, PRV-GFP was not seen in LDT or PPT cholinergic neurons (Table 1).

**Figure 7:**
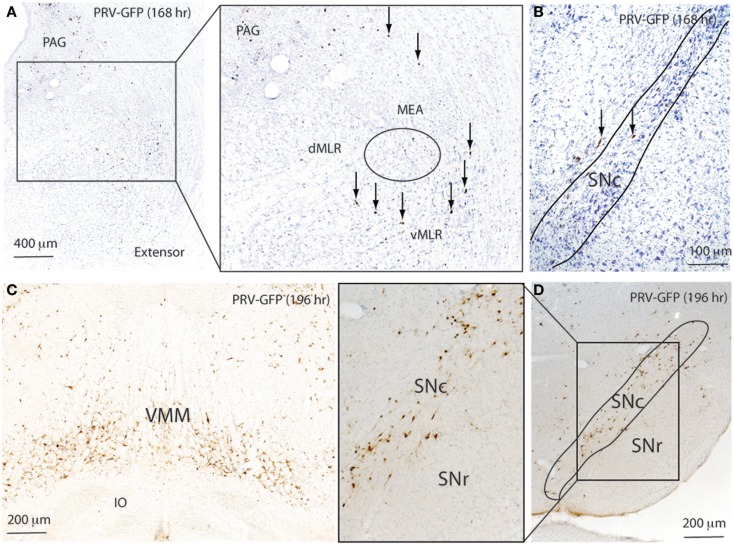
**Temporal and spatial distribution of PRV-GFP from the soleus (SOL; postural extensor)**. At 168 h after PRV-GFP injections in the SOL, PRV-GFP labels the PAG and vMLR and SNc **(A,B)**. PRV-GFP-ir neurons are more abundant in the VMM and SNc after 196 h **(C,D)**. These results suggest that for spinal motor control, the SNc- > vMLR projection may be more important for controlling extensor muscles (posture control), whereas the SNr- > MEA- > MLR projection may be more important for controlling flexor muscles, which are necessary for gait.

At 168 h after PRV injections in the extensor, a few PRV-GFP neurons were seen in the VMM. PRV-GFP appeared in the vMLR and SNc, but not in the MEA. At 196 h, more PRV-ir neurons were seen in the SNc but not in the SNr (Figure 7; Table 1), and PRV was highly expressed in the VMM, vlPAG, and MLR. However, PRV was not seen in the MEA (Figure 7; Table 1). At 196 h, PRV expression accumulated further in these regions. In addition, PRV was seen in the MEA, and some LDT cholinergic neurons, but not in PPT cholinergic neurons.

**Figure 8:**
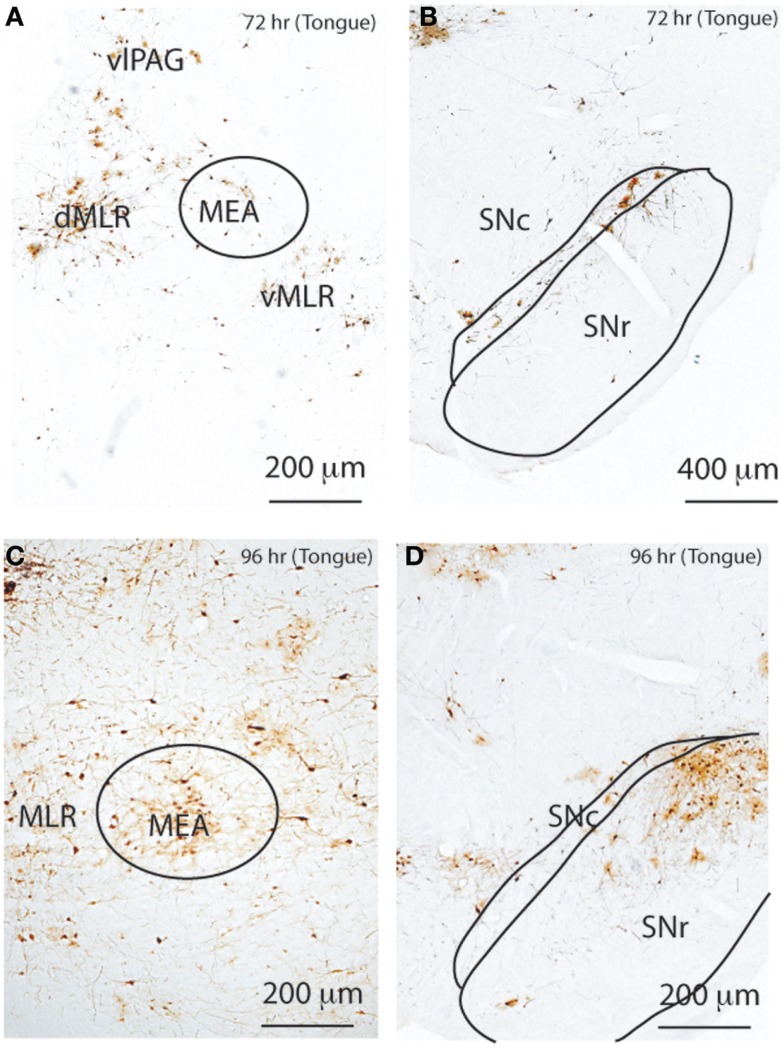
**Temporal and spatial distribution of PRV-GFP from the tongue**. At 72 h after PRV-GFP injection, the MLR **(A)** and SNc **(B)** show PRV-GFP-ir neurons ipsilateral to the injection site. After 96 h, the labeling is more extensive **(C,D)**, now including the MEA **(C)** and SNr **(D)**. The temporal sequence of PRV labeling suggests that the SNc- > MLR pathway requires less time for labeling than the SNr- > MEA- > MLR pathway following tongue injections.

To determine the neural connectivity of the MLR in the cranial motor system, we injected PRV-GFP into the tongue, which has both flexor (retraction) and extensor (protrusion) muscles, and perfused rats after 72 h (*n* = 2) and 96 h (*n* = 2), respectively. Rats perfused after 72 h showed PRV-GFP labeled neurons in the entire MLR region and SNc as well as RRF (Figure 8); however, no PRV-ir cells were seen in the MEA or SNr. By 96 h, there was substantial PRV-GFP labeling of cells not only in the MLR, but also in the MEA, SNr, and SNc as well (Figure 8). We also observed many PRV-GFP neurons in the vlPAG, RVM, and VMM.

Taken together, these temporal patterns of PRV-GFP labeling in the pontine motor circuitry are consistent with flexor and extensor muscles being influenced by two separate neural circuits: (1) an extensor circuit that regulates posture (maintained mainly by extensors in the limbs) involving a inhibitory input from the SNc (GABAergic and dopaminergic neurons) to the ventral MLR, which then regulates posture; and (2) a flexor circuit, necessary for locomotion and performance of specific tasks, which involves an inhibitory projection from the GABAergic SNr to the MEA, mainly to the dorsal MLR. The MLR also regulates cranial motor behavior.

## Discussion

The present study provides evidence that spinal-projecting neurons of the pontine tegmentum form, at least in part, the neuroanatomic basis of the MLR. This neuronal population specifically comprises reticulospinal glutamatergic cells that express high levels of the OX2 receptor. Consistent with this interpretation, bilateral chemical stimulation of the dorsal or ventral MLR neurons induced rhythmic walking and erect posture, respectively, whereas cell-specific lesions produced cataplexy and immobility. Our study also reveals a potential critical role for the MLR as a link between basal ganglia output and spinal cord motor circuits. Collectively, these results provide a new perspective for the neuroanatomic basis of the MLR as well as providing a context for understanding motor disturbances associated with cataplexy and PD.

### MLR control of motor behavior

The MLR was originally defined on the basis of electrical stimulation experiments, and hence its cellular identity was not well defined. Garcia-Rill and colleagues attempted to map sites that produced locomotion at low threshold, and found that the sites were located close to the cholinergic PPT (31, 32). Because PPT cholinergic neurons do not project directly to the spinal cord, the same investigators hypothesized that these neurons might influence locomotion by means of its projections to the ventral medulla (33). Skinner, Garcia-Rill, and colleagues found that injection of cholinergic agonist into the rostral ventromedial medulla (RVM, at the level of the facial nucleus) induced stepping behavior (25). However, the latency of agonist-induced stepping was long (5–10 min) and the duration short (5–10 s), which raises the possibility that stepping may have been triggered by a distant site controlled by the RVM. Consistent with this interpretation, our PRV-based transneuronal labeling revealed that PPT cholinergic neurons do not strongly project to the medulla. Interestingly, NMDA agonist in the RVM induced stepping with a short latency and long duration (1–5 min) (25), suggesting the possibility that glutamate inputs, possibly from the LPT, to the ventral medulla may be involved in regulation of locomotion. On the other hand, we found that lesions of the ventral medulla did not block locomotor activity or Fos immunoreactivity in spinal motor neurons induced by chemical stimulation of the MLR. Paradoxically, Lai, Siegel, and colleagues have consistently demonstrated that injections of cholinergic and glutamate agonists into the ventral medulla induce atonia (27, 34–36). Chemogenetic activation of the RVM in rats also does not promote motor behavior (Chen, Fuller, and Lu, unpublished observations). Finally, cell body lesions of the ventral medulla do not produce cataplexy or immobility attacks (37). Thus, while the foregoing data, taken together, do not provide conclusive evidence that glutamate inputs from the LPT (or other supra spinal sites) are necessary for locomotion, they do suggest that the structural integrity of the ventral medulla is not required for the MLR to regulate motor behavior and, moreover, that the ventral medulla is more critically involved in the regulation of muscle tone control than locomotion.

It should be noted that since we did not specifically measure stepping behavior on a treadmill, we could not rule out the possibility that PPT cholinergic neurons are uniquely involved in the regulation of stepping behavior. Furthermore, our study did not directly examine the role of PPT cholinergic neurons in locomotion nor does our experimental approach (including the lesions and stimulation experiments) allow us to exclude a contribution of PPT cholinergic neurons to locomotor control. We anticipate that the specific role and contribution of PPT cholinergic neurons in locomotion will be informed in future investigations using optogenetic and chemogenetic approaches. Our data do, however, continue to strongly support the concept that a population of glutamatergic spinally projecting neurons within the LPT, situated directly medially adjacent to the PPT, comprise a major structural element of the MLR. Additional and strong evidence in support of our hypothesis derives from our recent work, in which we performed extracellular recordings on spinally projecting neurons, verified by antidromic response to spinal cord stimulation, in the LPT of freely moving rats. In that we study, we demonstrated that non-cholinergic LPT neurons fired in bursts that were specifically associated with motor behavior, while non-spinally projecting neurons, including presumptive cholinergic PPT neurons, did not fire in association with motor behavior (12).

The anatomical results of the present study also suggest that spinally projecting neurons in the LPT may be the long-sought target of the descending projection of the basal ganglia. The descending pallidal and nigral descending projection was originally identified by Nauta and Mehler (38) as innervating the PPT, and this concept has been promulgated in many textbooks. Rather, and as Rye and colleagues have shown, the descending output of the SNr targets the MEA, (29) not the PPT, at least in rats. The MEA consists of small sized neurons, which are possibly local interneurons, but their targets have previously been unknown. Our transneuronal data indicate that spinally projecting neurons in the MLR are likely a major target of the MEA. Our results divide the spinally projecting neurons in the LPT into a ventral population involved in postural control, whose hypoactivity is associated with the postural collapse of cataplexy, and a dorsal population involved in locomotor facilitation, whose hypoactivity is linked to immobile attacks in rats. These structures are influenced by disynaptic input from the SNr and GPi, which are relayed through the MEA. According to our proposed model of MLR function (Figure 1F), basal ganglia output directs MLR activity patterns in accordance with a selected motor program, conveying information as to which muscles require activation to move (via the dMLR) and which muscles require activation to maintain postural stability (via the vMLR). Striatal (direct) and pallidal (indirect) output would therefore inhibit the SNr, and thereby disinhibit appropriate portions of the MEA, allowing for dMLR and vMLR activation and facilitating locomotion and postural enhancement, respectively, in the appropriate muscle groups.

Our model would further predict that, through its axonal projections to the spinal cord, the MLR provides excitatory input to motor pattern generator neurons or to motor neurons or to both. The degree to which this is a direct projection or one that utilizes either spinal or ventromedial medullary interneurons in intact animals remains an important subject for future study. In addition, our understanding of the relationship between the MLR and spinal cord circuitry is far from complete, and further study is necessary to determine physical correlates of postural and locomotor control at the spinal cord level. Although we did not focus on MLR control of the cranial motor system, what we have shown is consistent with previous work in which injections of PRV were placed in the tongue (39). In addition, we found that chemical stimulation of the MLR induced vocalization and whisker movements in rats.

### MLR lesions cause cataplexy and akinesia

In further support of a role for MLR neurons in the control of muscle tone, we have observed that lesions of the MLR region facilitate cataplectic behavior. As the lesioned neurons express OX2 receptor mRNA, this finding links to the established role of orexin signaling in cataplexy, including the loss of orexin signaling in orexin knockout mice (40), dogs with OX2 receptor mutations (41), and human patients lacking orexin neurons (42). During short cataplectic attacks (under 30 s), the EEG of our rats did not change from the waking pattern; however, in many longer bouts, the EEG gradually transitioned into a slow pattern indicative of a non-REM sleep state. Cataplexy was particularly prominent after lesions of the vMLR. Many of the vMLR neurons express OX2 receptors, which may, in part, explain why orexin deficiency would also make animals prone to cataplexy. Of note, PPT cholinergic neurons express OX1 receptors, which unlike OX2 receptors are not associated with cataplexy. On the other hand, lesions targeting the dMLR resulted in immobility attacks. The animals appeared unable to move during the attacks, which is consistent with the concept that the dMLR promotes locomotion. These findings are consistent with the idea that the MLR comprise two divisions, a dorsal group involved in locomotor facilitation and a ventral group involved in postural regulation.

### MLR and parkinsonism

The immobility attacks seen in our rats with dMLR lesions are reminiscent of the gait freezing observed in PD. These observations suggest that in patients with PD, these same underlying neural circuits may contribute to akinesia by transmitting inhibitory influences from hyperactive SNr neurons to the dMLR or possibly through lesions in the dMLR in PD (43). Our study has also shown that the vMLR is critically linked to postural muscle tone control, and this circuitry could contribute to rigidity in PD patients, through loss of SNc GABAergic neurons, which may be necessary to break extensor tone. In the past, loss of SNc neurons has focused on dopaminergic neurons, but it is known that there is a large GABAergic population intermixed with the dopaminergic neurons, and their role has received relatively scant attention. On the other hand, alpha-synuclein deposits in the pons precede those in the substantia nigra (44, 45), so it is possible that the MLR and/or MEA neurons may be damaged independently, and earlier in disease progression, to produce akinesia and posture instability. Again, the roles of these different cellular populations in PD require further study.

Patients with PD also have cranial motor symptoms, including a mask-like facial appearance, low volume speech, and difficulty swallowing. Our results indicate that the MLR region may also contribute to cranial nerve motor control, and that manipulations such as DBS in this region may also be effective for treating orofacial dysfunction of PD. Indeed, the entire basis for the effects of DBS in the PPT region may require a re-evaluation in terms of the type of neurons responsible for improvement in akinesia and gait that have been observed in both PD primate models and patients with PD (24, 46–48). In particular, a more detailed understanding of how basal ganglia influence the activity of the MLR may greatly inform the design of future treatments for PD.

## Materials and Methods

### Animals

Pathogen-free, adult, male Sprague-Dawley rats (275–300 g, Harlan) were individually housed and had free access to food and water. All animals were housed under controlled conditions (12 h light starting at 07:00 a.m., 100 lux) in an isolated ventilated chamber maintained at 20–22°C. All protocols were approved by the Institutional Animal Care and Use Committee of Beth Israel Deaconess Medical Center.

### Physiology

#### EEG/EMG Lead Implantation and Sleep Analysis

Rats were anesthetized with chloral hydrate (350 mg/kg), and the skull exposed. Four EEG screw electrodes were implanted into the skull, in the frontal (2) and parietal bones (2) of each side, and two flexible EMG wire electrodes were placed into the neck muscles. The free ends of the leads were soldered into a socket that was attached to the skull with dental cement, and the incision was closed using wound clips. One week after surgery, the sockets were connected via flexible recording cables and a commutator to a Grass polygraph and computer. Signals were digitized by a PC running *Sleepsign* recording (Kissei, Japan).

Three (Wake, NREM, and REM sleep) behavioral states were visually identified and analyzed in 10 s epochs for daily sleep-wake cycle analysis. The behavioral states were classified in wake, characterized by high frequency, low-voltage EEG signals coupled with high levels of EMG activity EG; NREM sleep (NR) was characterized by high-amplitude, low-frequency EEG signals, and in the absence of motor activity; REM sleep was characterized by low-amplitude, high-frequency theta-like EEG activity, and muscle atonia.

### Histology and tracing

#### Stereotaxic Injections of Tracers, OX-SAP

Under chloral hydrate anesthesia (7% in saline, 350 mg/kg), a fine glass pipette containing the tracers (1.0% Cholera toxin subunit B (CTB, List Biological), FG (5%), orexin-saporin (Advanced Targeting Systems) was lowered to the pre-calculated targets. Injections of 3–100 nl volumes were made over 10 min by an air pressure system. After two additional minutes, the pipette was slowly withdrawn and the incision was closed with wound clips. Animals survived for 7 days. Coordinates: LPT, AP = −7.6 mm, ML = 1.6 mm, DV = 7.2 mm; VRM, AP = −11.9 mm, ML = 0.0 mm, DV = 8.0 mm. FG was injected at T1 level.

#### PRV-GFP Injection

PRV-GFP-152 was the generous gift of Dr. Lynn Enquist (Princeton University, Princeton, NJ, USA). Individual aliquots were frozen at −80°C and thawed immediately before the injection. Animals were anesthetized with choral hydrate, and injections of 30 μl (10^9^ pfu/ml) were placed either into the tongue muscles of right side or the leg muscles of right side, by using a 50 μl Hamilton syringe. In the leg injections, the right leg was shaved and the muscle groups were exposed, allowing injection of PRV-GFP into the soleus (extensor) or tibialis anterior (flexor) muscles under direct observation.

#### Ibotenic Acid Stimulation

For fast recovery from anesthesia, we anesthetized rats with isoflurane and injected 3 nl of ibotenic acid (10%) by air pressure in either the dorsal or ventral LPT (see above for coordinates). Wounds were closed with wound clips and rats woke up around 5 min after the surgery.

#### Perfusion

Animals were deeply anesthetized by chloral hydrate (500 mg/kg), then perfused with 50 ml saline, followed by 500 ml 10% formalin through the heart. The brains were removed, post-fixed for 4 h in 10% formalin, and then equilibrated in 20% sucrose in PBS overnight.

#### Immunohistochemistry

The brains were sectioned on a freezing microtome at 40 μm into four series. Sections were washed in 0.1M phosphate-buffered saline, pH 7.4 (PBS, two changes), and then incubated in the primary antiserum (rabbit polycolonal antibody agonist c-Fos, 1: 150,000, AB5, Oncogene Sciences; polycolonal goat antibody against ChAT, 1:2000, AB144, Chemicon); goat anti- CTB, 1:100,000, catalog # 114, List Biological; rat anti-GFP 1:5000, catalog# 69050, Novagen) for 1 day at room temperature. The c-Fos antiserum was raised against a synthetic peptide representing residues 4–17 from human c-Fos. The ChAT antiserum was raised against human placental enzyme and recognized a single band of about 62 kD m.w. on Western blots of rat brain (manufacturer’s technical information). It stains a pattern of neuronal morphology and distribution in the rat mesopintine tegmentum consistent with previous reports (30). The CTB antibody was made against purified cholera toxin B subunit. It does not stain anything in uninjected brains. The GFP antiserum was raised against purified jellyfish GFP, and also does not stain anything in normal rat brains. Cre was raised against bacteriophage P1 Cre recombinase.

Sections were then washed in PBS and incubated in biotinylated secondary antiserum (against appropriate species IgG, 1:1,000 in PBS) for 1 h, washed in PBS, and incubated in avidin-biotin-horseradish peroxidase conjugate (Vector) for 1 h. Sections were then washed again and incubated in a 0.06% solution of 3,3-diaminobenzidine tetrahydrochloride (DAB, Sigma) plus 0.02% H_2_O_2._ The sections were stained brown with DAB only or black by adding 0.05% cobalt chloride and 0.01% nickel ammonium sulfate to the DAB solutions.

For immunohistochemical controls for the secondary antibody, primary antibodies were omitted and the tissue showed no immunoreactivity above background.

#### *In situ* hybridization of VGLUT2 mRNA, D2 mRNA, and OX2 mRNA

This was done as in Marcus et al. (13). Sections were prepared as above for immunostaining (CTB, Fos, or FG), except that only DEPC treated solutions were used, and serum was omitted from incubations. Sections were then acetylated and hybridized overnight (55°C) with a ^35^S-labeled cRNA probe synthesized from a plasmid containing VGLUT2 (1615–1979 of GenBank entry AF271235), OX2 receptor (Dr. Marcus, Harvard Medical School), or D2 dopamine receptor (gift from Dr. Weaver at UMASS, Worcester, MA, USA). After a succession of 1 h washes (2×SSC/1 mM DTT, 50°C; 0.2×SSC/1 mM DTT, 55°C; 0.2×SSC/1 mM DTT, 60°C), the tissue was treated with RNase-A (Boehringer-Mannheim, Indianapolis) and washed under conditions of increasing stringency, including a 30 min wash at 60°C in 0.1% SSC. The tissue was then dehydrated in alcohols and air-dried. Slides were developed in Kodak D-19, fixed, and then dehydrated, and coverslipped.

## Conflict of Interest Statement

The authors declare that the research was conducted in the absence of any commercial or financial relationships that could be construed as a potential conflict of interest.

## References

[1] Rhines R Magoun HW Retromammillary inhibition of cortically induced ­movement. Proc Soc Exp Biol Med (1946) 63:76–8. 10.3181/00379727-63-15500 20274270

[2] Sprague JM Chambers WW Control of posture by reticular formation and cerebellum in the intract, anesthetized and unanesthetized and in the decerebrated cat. Am J Physiol (1954) 176:52–64. 13124495 10.1152/ajplegacy.1953.176.1.52

[3] Shik ML Severin FV Orlovskii GN [Control of walking and running by means of electric stimulation of the midbrain]. Biofizika (1966) 11:659–66. 6000625

[4] Berman AL The Brain Stem of the Cat; A Cytoarchitectonic Atlas with Stereotaxic Coordinates. Madison: University of Wisconsin Press (1968).

[5] Skinner RD Kinjo N Henderson V Garcia-Rill E Locomotor projections from the pedunculopontine nucleus to the spinal cord. Neuroreport (1990) 1:183–6. 10.1097/00001756-199011000-00008 2129877

[6] Sinnamon HM Ginzburg RN Kurose GA. Midbrain stimulation in the anesthetized rat: direct locomotor effects and modulation of locomotion produced by hypothalamic stimulation. Neuroscience (1987) 20:695–707. 10.1016/0306-4522(87)90120-5 3587613

[7] Saper CB Swanson LW Cowan WM. An autoradiographic study of the efferent connections of the lateral hypothalamic area in the rat. J Comp Neurol (1979) 183:689–706. 10.1002/cne.901830402 105019

[8] Rye DB Lee HJ Saper CB Wainer BH. Medullary and spinal efferents of the pedunculopontine tegmental nucleus and adjacent mesopontine tegmentum in the rat. J Comp Neurol (1988) 269:315–41. 10.1002/cne.902690302 2453532

[9] Lu J Sherman D Devor M Saper CB. A putative flip-flop switch for control of REM sleep. Nature (2006) 441:589–94. 10.1038/nature04767 16688184

[10] Skinner RD Kinjo N Ishikawa Y Biedermann JA Garcia-Rill E. Locomotor projections from the pedunculopontine nucleus to the medioventral medulla. Neuroreport (1990) 1:207–10. 10.1097/00001756-199011000-00008 2129882

[11] Sukhotinsky I Hopkins DA Lu J Saper CB Devor M. Movement suppression during anesthesia: neural projections from the mesopontine tegmentum to areas involved in motor control. J Comp Neurol (2005) 489:425–48. 10.1002/cne.20636 16025457

[12] Thankachan S Fuller PM Lu J. Movement- and behavioral state-dependent activity of pontine reticulospinal neurons. Neuroscience (2012) 221:125–39. 10.1016/j.neuroscience.2012.06.069 22796072 PMC3424299

[13] Marcus JN Aschkenasi CJ Lee CE Chemelli RM Saper CB Yanagisawa M Differential expression of orexin receptors 1 and 2 in the rat brain. J Comp Neurol (2001) 435:6–25. 10.1002/cne.1190 11370008

[14] Lee MG Hassani OK Jones BE. Discharge of identified orexin/hypocretin neurons across the sleep-waking cycle. J Neurosci (2005) 25:6716–20. 10.1523/JNEUROSCI.1887-05.2005 16014733 PMC6725432

[15] Mileykovskiy BY Kiyashchenko LI Siegel JM. Behavioral correlates of activity in identified hypocretin/orexin neurons. Neuron (2005) 46:787–98. 10.1016/j.neuron.2005.04.035 15924864 PMC8281334

[16] Takakusaki K Takahashi K Saitoh K Harada H Okumura T Kayama Y Orexinergic projections to the cat midbrain mediate alternation of emotional behavioural states from locomotion to cataplexy. J Physiol (2005) 568:1003–20. 10.1113/jphysiol.2005.085829 16123113 PMC1464186

[17] Sakurai T Amemiya A Ishii M Matsuzaki I Chemelli RM Tanaka H Orexins and orexin receptors: a family of hypothalamic neuropeptides and G protein-coupled receptors that regulate feeding behavior. Cell (1998) 92:573–85. 10.1016/S0092-8674(00)80949-6 9491897

[18] D’Cruz OF Vaughn BV Gold SH Greenwood RS. Symptomatic cataplexy in pontomedullary lesions. Neurology (1994) 44:2189–91. 10.1212/WNL.44.11.2189 7969983

[19] Fernandez JM Sadaba F Villaverde FJ Alvaro LC Cortina C Cataplexy associated with midbrain lesion. Neurology (1995) 45:393–4. 10.1212/WNL.45.2.393-a 7854550

[20] Mathis J Hess CW Bassetti C. Isolated mediotegmental lesion causing narcolepsy and rapid eye movement sleep behaviour disorder: a case evidencing a common pathway in narcolepsy and rapid eye movement sleep behaviour disorder. J Neurol Neurosurg Psychiatry (2007) 78:427–9. 10.1136/jnnp.2006.099515 17369596 PMC2077786

[21] Plazzi G Montagna P Provini F Bizzi A Cohen M Lugaresi E Pontine lesions in idiopathic narcolepsy. Neurology (1996) 46:1250–4. 10.1212/WNL.46.5.1250 8628461

[22] Stahl SM Layzer RB Aminoff MJ Townsend JJ Feldon S. Continuous cataplexy in a patient with a midbrain tumor: the limp man syndrome. Neurology (1980) 30:1115–8. 10.1212/WNL.30.10.1115 6252510

[23] Blandini F Nappi G Tassorelli C Martignoni E Functional changes of the basal ganglia circuitry in Parkinson’s disease. Prog Neurobiol (2000) 62:63–88. 10.1016/S0301-0082(99)00067-2 10821982

[24] Stefani A Lozano AM Peppe A Stanzione P Galati S Tropepi D Bilateral deep brain stimulation of the pedunculopontine and subthalamic nuclei in severe Parkinson’s disease. Brain (2007) 130:1596–607. 10.1093/brain/awl346 17251240

[25] Kinjo N Atsuta Y Webber M Kyle R Skinner RD Garcia-Rill E. Medioventral medulla-induced locomotion. Brain Res Bull (1990) 24:509–16. 10.1016/0361-9230(90)90104-8 2186847

[26] Paxinos G Watson C The Rat Brain in Stereotaxic Coordinates. 6th ed Amsterdam; Boston: Academic Press/Elsevier (2007).

[27] Lai YY Kodama T Schenkel E Siegel JM. Behavioral response and transmitter release during atonia elicited by medial medullary stimulation. J Neurophysiol (2010) 104:2024–33. 10.1152/jn.00528.2010 20668280 PMC2957456

[28] Fuller P Sherman D Pedersen NP Saper CB Lu J. Reassessment of the structural basis of the ascending arousal system. J Comp Neurol (2011) 519:933–56. 10.1002/cne.22559 21280045 PMC3119596

[29] Rye DB Saper CB Lee HJ Wainer BH. Pedunculopontine tegmental nucleus of the rat: cytoarchitecture, cytochemistry, and some extrapyramidal connections of the mesopontine tegmentum. J Comp Neurol (1987) 259:483–528. 10.1002/cne.902590403 2885347

[30] Lee HJ Rye DB Hallanger AE Levey AI Wainer BH. Cholinergic vs. noncholinergic efferents from the mesopontine tegmentum to the extrapyramidal motor system nuclei. J Comp Neurol (1988) 275:469–92. 10.1002/cne.902750402 2461392

[31] Garcia-Rill E Houser CR Skinner RD Smith W Woodward DJ. Locomotion-inducing sites in the vicinity of the pedunculopontine nucleus. Brain Res Bull (1987) 18:731–8. 10.1016/0361-9230(87)90208-5 3304544

[32] Garcia-Rill E Skinner RD Fitzgerald JA Chemical activation of the mesencephalic locomotor region. Brain Res (1985) 330:43–54. 10.1016/0006-8993(85)90006-X 3986540

[33] Garcia-Rill E Skinner RD The mesencephalic locomotor region. II. Projections to reticulospinal neurons. Brain Res (1987) 411:13–20. 10.1016/0006-8993(87)90676-7 3607422

[34] Hajnik T Lai YY Siegel JM. Atonia-related regions in the rodent pons and medulla. J Neurophysiol (2000) 84:1942–8. 11024087 10.1152/jn.2000.84.4.1942 PMC8841098

[35] Kodama T Lai YY Siegel JM. Enhancement of acetylcholine release during REM sleep in the caudomedial medulla as measured by in vivo microdialysis. Brain Res (1992) 580:348–50. 10.1016/0006-8993(92)90967-E 1504813 PMC9046437

[36] Lai YY Siegel JM. Medullary regions mediating atonia. J Neurosci (1988) 8:4790–6. 2904495 10.1523/JNEUROSCI.08-12-04790.1988 PMC6569564

[37] Vetrivelan R Fuller PM Tong Q Lu J. Medullary circuitry regulating rapid eye movement sleep and motor atonia. J Neurosci (2009) 29:9361–9. 10.1523/JNEUROSCI.0737-09.2009 19625526 PMC2758912

[38] Nauta WJ Mehler WR Projections of the lentiform nucleus in the monkey. Brain Res (1966) 1:3–42. 10.1016/0006-8993(66)90103-X 4956247

[39] Fay RA Norgren R. Identification of rat brainstem multisynaptic connections to the oral motor nuclei using pseudorabies virus. III. Lingual muscle motor systems. Brain Res Brain Res Rev (1997) 25:291–311. 10.1016/S0165-0173(97)00026-X 9495560

[40] Chemelli RM Willie JT Sinton CM Elmquist JK Scammell T Lee C Narcolepsy in orexin knockout mice: molecular genetics of sleep regulation. Cell (1999) 98:437–51. 10.1016/S0092-8674(00)81973-X 10481909

[41] Lin L Faraco J Li R Kadotani H Rogers W Lin X The sleep disorder canine narcolepsy is caused by a mutation in the hypocretin (orexin) receptor 2 gene. Cell (1999) 98:365–76. 10.1016/S0092-8674(00)81965-0 10458611

[42] Nishino S Ripley B Overeem S Lammers GJ Mignot E Hypocretin (orexin) deficiency in human narcolepsy. Lancet (2000) 355:39–40. 10.1016/S0140-6736(99)05582-8 10615891

[43] Braak H Rub U Gai WP Del Tredici K. Idiopathic Parkinson’s disease: possible routes by which vulnerable neuronal types may be subject to neuroinvasion by an unknown pathogen. J Neural Transm (2003) 110:517–36. 10.1007/s00702-002-0808-2 12721813

[44] Braak H Braak E Yilmazer D Schultz C de Vos RA Jansen EN Nigral and extranigral pathology in Parkinson’s disease. J Neural Transm Suppl (1995) 46:15–31. 8821039

[45] Braak H Del Tredici K Invited article: nervous system pathology in sporadic Parkinson disease. Neurology (2008) 70:1916–25. 10.1212/01.wnl.0000312279.49272.9f 18474848

[46] Nandi D Aziz TZ Giladi N Winter J Stein JF. Reversal of akinesia in experimental parkinsonism by GABA antagonist microinjections in the pedunculopontine nucleus. Brain (2002) 125:2418–30. 10.1093/brain/awf259 12390969

[47] Nandi D Aziz TZ Liu X Stein JF. Brainstem motor loops in the control of movement. Mov Disord (2002) 17(Suppl 3):S22–7. 10.1002/mds.10139 11948752

[48] Nandi D Liu X Winter JL Aziz TZ Stein JF. Deep brain stimulation of the pedunculopontine region in the normal non-human primate. J Clin Neurosci (2002) 9:170–4. 10.1054/jocn.2001.0943 11922707

